# Association of rs4977574 with Lipid Phenotypes, Smoking Status, and Statin Exposure in a Saudi Cardiovascular Cohort: A Sensitivity-Adjusted Genetic Association Study

**DOI:** 10.3390/jcm15135237

**Published:** 2026-07-04

**Authors:** Neda M. Bogari, Hind Mansour Naffadi, Lujain Ibrahim Essa, Amr A. Amin, Rami Obaid, Reem M. Allam

**Affiliations:** 1 Department of Medical Genetics, Faculty of Medicine, Umm Al-Qura University, Makkah 21955, Saudi Arabia; nmbogari@uqu.edu.sa (N.M.B.); hmnaffadi@uqu.edu.sa (H.M.N.); ph.lujain.essa@gmail.com (L.I.E.); 2 Department of Medical Biochemistry, Faculty of Medicine, Umm Al-Qura University, Makkah 21955, Saudi Arabia; aaamin@uqu.edu.sa; 3 Faculty of Medicine, Ain-Shams University, Cairo 11566, Egypt; 4 Department of Medical Genetics, Faculty of Medicine at Al-Qunfudah, Umm Al-Qura University, Al-Qunfudah 21912, Saudi Arabia; raaobaid@uqu.edu.sa; 5 Department of Clinical Pathology, Faculty of Medicine, Zagazig University, Zagazig 44519, Egypt

**Keywords:** rs4977574, 9p21, CAD, HDL-c, triglycerides, atorvastatin

## Abstract

**Background**: Coronary artery disease (CAD) arises from the convergence of genetic susceptibility, lipid dysregulation, and modifiable environmental exposures. The polymorphism rs4977574, located proximal to the CDKN2A/CDKN2B gene cluster, has been repeatedly implicated in CAD risk across several populations, yet its relationship to intermediate cardiometabolic phenotypes and pharmacological treatment patterns in Saudi individuals remains poorly characterized. **Objective**: This study aimed to evaluate the association of rs4977574 with CAD status, lipid-related phenotypes, smoking history, obesity, and atorvastatin exposure in a Saudi cardiovascular cohort, and to assess the robustness of observed associations through sensitivity-adjusted analyses excluding participants with major metabolic confounders. **Methods**: A case–control genetic association study was conducted in Saudi participants with clinically confirmed CAD and healthy controls. Genomic DNA was genotyped for rs4977574 using TaqMan^®^ allelic discrimination assays. Genotype–phenotype associations were examined using chi-square testing, binary logistic regression under additive and dominant inheritance models, and one-way ANOVA for continuous lipid traits. Hardy–Weinberg equilibrium (HWE) was assessed in controls. Sensitivity analyses were conducted by sequentially excluding participants with obesity, smoking, diabetes mellitus, hypertension, dyslipidaemia, and statin use. **Results**: After covariate adjustment, rs4977574 was not independently associated with CAD case–control status under any inheritance model. Genotype-stratified analyses identified significant differences in HDL-cholesterol and triglyceride concentrations among cases, with no equivalent effects on total cholesterol or LDL-cholesterol. A significant association was observed between rs4977574 genotype and atorvastatin prescribing patterns. Sensitivity-adjusted analyses were directionally consistent with primary findings. HWE deviation persisted in controls after sequential metabolic exclusions, implicating population stratification or regional genetic heterogeneity rather than sample selection bias. **Conclusions:** Although rs4977574 did not associate independently with CAD susceptibility, its relationship with HDL-cholesterol, triglycerides, and atorvastatin exposure indicates that this locus contributes to cardiometabolic phenotypic heterogeneity in this Saudi cohort. These findings support phenotype-oriented and pharmacogenetically informed approaches in regional cardiovascular genetics and highlight the need for larger, ancestry-stratified investigations across Middle Eastern populations.

## 1. Introduction

Coronary artery disease (CAD) continues to rank among the foremost contributors to premature death and disability across the globe, placing a disproportionate strain on health systems in the Middle East, including Saudi Arabia [1,2]. Over recent decades, epidemiological transitions within the region have driven sharp increases in the prevalence of obesity, type 2 diabetes mellitus, dyslipidemia, tobacco use, and sedentary behavior—a convergence of risk factors that have amplified the cardiovascular disease burden considerably. Alongside these behavioral and metabolic determinants, heritable variation in the human genome is now well established as a meaningful source of interindividual differences in cardiovascular susceptibility, disease trajectory, and pharmacological response [3]. Translating that genetic knowledge into practical risk stratification tools and precision medicine frameworks therefore represents one of the more pressing priorities in contemporary preventive cardiology [4].

Among the chromosomal regions most consistently implicated in CAD across diverse populations, the 9p21 locus has attracted sustained investigative interest. Within this region, the single-nucleotide polymorphism rs4977574—situated in close proximity to the cyclin-dependent kinase inhibitor 2A and 2B (CDKN2A/CDKN2B) gene cluster—has emerged as one of the more reproducible genetic signals in large-scale association analyses [5,6]. Genome-wide association studies and subsequent meta-analyses spanning tens of thousands of participants have collectively demonstrated that this variant associates with susceptibility to myocardial infarction, coronary atherosclerosis, and related adverse outcomes. The biological pathways through which 9p21 exerts its influence remain the subject of ongoing enquiry; current evidence points to roles in vascular smooth muscle proliferation, endothelial dysfunction, inflammatory signaling, and the regulation of cellular senescence through the CDKN2A/2B axis. Despite this mechanistic progress, the strength of association between rs4977574 and overt cardiovascular disease varies substantially across ancestral groups, cautioning against uncritical extrapolation of European-derived effect estimates to non-European populations [7].

A notable shift has occurred in the orientation of cardiovascular genetic research over the past decade. Earlier investigations concentrated predominantly on binary disease outcomes, seeking to confirm or refute whether a given variant conferred susceptibility to manifest coronary events. More recent work has moved toward intermediate cardiometabolic phenotypes—lipid concentrations, inflammatory markers, insulin resistance, body composition—and toward gene–environment interaction analyses that account for modifiable exposures [6]. This reorientation reflects an appreciation that genetic variants may exert metabolically meaningful effects that precede, or operate independently of, clinical disease. In this framework, examining rs4977574 in relation to plasma lipid profiles, smoking history, obesity indices, and patterns of statin exposure could yield clinically informative results even when direct associations with incident coronary events are modest or heterogeneous. Understanding whether this variant modulates lipid-related phenotypes or interacts with tobacco exposure and pharmacological lipid lowering has direct relevance to individualized cardiovascular risk assessment [8,9].

Statins are the pharmacological cornerstone of cardiovascular risk reduction, supported by a robust evidence base spanning primary and secondary prevention settings. Nevertheless, clinicians routinely encounter wide variation in lipid-lowering response, residual vascular risk after treatment initiation, and tolerability profiles among patients prescribed these agents [10]. Genetic determinants of statin pharmacokinetics and pharmacodynamics are increasingly recognized, with prior research focusing on variants in genes governing drug transport, hepatic uptake, and cholesterol synthesis [11]. Whether susceptibility loci such as rs4977574—not directly involved in lipid metabolism pathways—nonetheless influence the likelihood of statin exposure or shape the residual cardiometabolic phenotype in treated patients represents a less examined but clinically pertinent question [12]. Addressing it requires study designs that move beyond dichotomous case–control comparisons toward stratified analyses that accommodate pharmacological exposures and metabolic covariates.

The Saudi Arabian population offers a scientifically important but underrepresented context for this line of inquiry. A high background prevalence of cardiometabolic disease, combined with a population genetic architecture shaped by historical consanguinity, regional admixture, and relative isolation from admixture with other continental populations, means that allele frequencies and linkage disequilibrium patterns at the 9p21 locus may differ materially from those reported in predominantly European or East Asian cohorts [13]. These differences carry practical implications: risk effect sizes derived from large international consortia cannot be assumed to apply with equal validity in Saudi patients. Locally conducted investigations are therefore not merely confirmatory exercises but are necessary for establishing population-specific genotype–phenotype relationships that can inform clinical decision-making in this setting [14].

Notwithstanding the breadth of the international literature on chromosome 9p21 variants, published data specifically addressing rs4977574 in Saudi cardiovascular cohorts remain limited. In particular, few regional studies have examined the relationship between this polymorphism and lipid phenotypes, smoking status, obesity, or statin exposure, and fewer still have incorporated sensitivity analyses designed to isolate genotype effects from the confounding influence of major metabolic comorbidities. This evidence gap is consequential, because uncontrolled metabolic heterogeneity within study samples can attenuate or inflate apparent genotype associations and undermine the clinical interpretability of findings.

The present study was designed to address these gaps. Using data from a Saudi cardiovascular cohort, we investigated the association of rs4977574 with coronary artery disease status, a panel of lipid-related phenotypes, smoking exposure, obesity classification, and statin use. Sensitivity-adjusted analyses were additionally performed by excluding participants with substantial metabolic confounders, enabling a more refined appraisal of the genotype–phenotype relationships under conditions of reduced cardiometabolic heterogeneity. Together, these analyses were intended to clarify whether rs4977574 contributes to cardiometabolic variability within the Saudi population beyond its established role as a susceptibility variant for overt coronary disease.

## 2. Materials and Methods

### 2.1. Study Design and Ethical Considerations

This investigation was conducted as a case–control genetic association study nested within a Saudi cardiovascular cohort. Ethical approval was obtained from the relevant institutional review board prior to participant recruitment, and all procedures were performed in accordance with the principles of the Declaration of Helsinki. Written informed consent was secured from each participant before enrolment, and data were handled in accordance with applicable data protection regulations. The reporting of methods and findings adheres to the STrengthening the REporting of Genetic Association Studies (STREGA) guidelines, an extension of the STROBE statement specifically adapted for genetic epidemiology research [15].

### 2.2. Participant Recruitment and Sample Collection

Participants were recruited from cardiology outpatient clinics and community health settings across the study region. Cases were defined as Saudi individuals with clinically confirmed coronary artery disease, established on the basis of documented myocardial infarction, angiographically verified coronary stenosis, or validated electrocardiographic and imaging evidence of ischemic heart disease. Controls were recruited from the same geographic catchment area and were confirmed to be free of known cardiovascular disease through structured clinical interviews, physical examination, and review of medical records. Exclusion criteria applied to both groups included chronic renal or hepatic disease, autoimmune conditions, active malignancy, pregnancy, and the use of medications known to substantially alter lipid metabolism independently of the study treatments of interest.

Venous blood samples were drawn from all participants under standardized phlebotomy conditions following an overnight fast of at least eight hours. Samples were collected into vacutainer tubes pre-filled with ethylenediaminetetraacetic acid (EDTA) as an anticoagulant. Following collection, tubes were labelled with anonymized participant identifiers, transported to the molecular laboratory on wet ice within two hours, and processed promptly to minimize pre-analytical variability. Aliquots designated for biochemical analysis were separated by centrifugation at 3000× *g* for 10 min at 4 °C, with resulting plasma stored at −80 °C pending lipid assays. The remaining whole-blood fraction retained in EDTA tubes was used directly for genomic DNA extraction.

### 2.3. Genomic DNA Extraction

Genomic DNA was isolated from whole-blood samples using the MagMAX™-96 Multi-Sample DNA Extraction Kit (Thermo Fisher Scientific, Waltham, MA, USA), a bead-based magnetic particle system compatible with high-throughput 96-well processing. All extraction steps were performed in strict accordance with the manufacturer’s validated protocol. Briefly, red blood cell lysis was achieved using the proprietary lysis buffer supplied with the kit, after which white blood cell pellets were subjected to proteinase K digestion to facilitate cell membrane disruption and protein denaturation. Magnetic beads coated with nucleic acid-binding chemistry were then introduced to selectively capture genomic DNA, and sequential wash steps employing ethanol-based buffers were applied to remove residual protein contaminants, haem derivatives, and PCR-inhibitory substances.

Following the final wash, bound DNA was eluted from the magnetic beads using a low-salt elution buffer, yielding genomic material suitable for downstream molecular applications. The eluate was resuspended in Tris–EDTA (TE) buffer at pH 8.0, a formulation chosen to stabilize double-stranded DNA and inhibit nuclease-mediated degradation during storage. All extracted DNA samples were stored at −20 °C in clearly labelled 1.5 mL microcentrifuge tubes until required for quality assessment and genotyping procedures. To minimize the risk of DNA fragmentation attributable to freeze–thaw cycling, samples were aliquoted at the point of extraction, with primary stocks retained at −20 °C and working aliquots used for genotyping.

### 2.4. DNA Quantity and Quality Assessment

The yield and purity of each extracted DNA sample were evaluated prior to genotyping using a NanoDrop™ 2000 spectrophotometer (Thermo Fisher Scientific, Wilmington, DE, USA). Absorbance measurements were recorded at wavelengths of 260 nm and 280 nm, and the A260/A280 ratio was used as the primary index of sample purity. Ratios within the range of 1.8 to 2.0 were accepted as indicative of adequately pure DNA, free from significant protein contamination; samples yielding ratios outside this range were subjected to re-extraction rather than proceeding to genotyping [16]. The A260/A230 ratio was additionally recorded as a secondary measure of contamination by organic compounds, chaotropic salts, or residual extraction reagents.

DNA integrity was confirmed through electrophoretic separation on 1.25% agarose gels prepared with Tris–borate–EDTA (TBE) running buffer. Samples were combined with loading dye and run alongside a 1 kb molecular weight ladder at 80 V for 45 min. Gels were stained with ethidium bromide and visualized under ultraviolet transillumination. High-molecular-weight banding without evidence of smearing or degradation products was required for a sample to be considered acceptable for downstream analysis. Samples in which gel electrophoresis indicated partial degradation were excluded from genotyping. Only samples meeting both spectrophotometric and electrophoretic quality thresholds were transferred in normalized concentrations into 96-well PCR plates and stored at −20 °C pending allelic discrimination.

### 2.5. Genotyping of Rs4977574 by TaqMan Allelic Discrimination

#### 2.5.1. Assay Design and Reagent Preparation

Genotyping of the rs4977574 single-nucleotide polymorphism (SNP), located within the chromosome 9p21.3 locus in the vicinity of the CDKN2A/CDKN2B gene cluster, was performed using a commercially validated TaqMan^®^ SNP Genotyping Assay (Applied Biosystems, Thermo Fisher Scientific, Foster City, CA, USA). The assay incorporates a pair of sequence-specific TaqMan^®^ MGB (minor groove binder) fluorescent probes, each labelled with a spectrally distinct fluorophore—VIC^®^ for the reference allele and FAM™ for the alternate allele—enabling simultaneous discrimination of both alleles within a single reaction well. MGB chemistry was selected for its capacity to confer enhanced probe melting temperature stability and superior allelic discrimination even in the presence of single-nucleotide mismatches, which is particularly advantageous for genotyping in populations where linkage disequilibrium patterns may deviate from reference panels.

Reaction master mixes were prepared in a dedicated pre-PCR area using filtered aerosol-resistant pipette tips to minimize the risk of cross-contamination. Each reaction well contained TaqMan^®^ Genotyping Master Mix (2×), the rs4977574-specific assay mix at the manufacturer-recommended working concentration (1× final concentration), and template DNA at a standardized input of 20 ng per reaction in a final volume of 10 μL. Reaction plates were sealed with optical adhesive film, briefly centrifuged to consolidate contents and eliminate air pockets, and immediately transferred to the thermal cycler.

#### 2.5.2. Thermal Cycling Conditions

Amplification and allele discrimination were carried out on a real-time PCR system (Applied Biosystems, Thermo Fisher Scientific, Foster City, CA, USA) programmed with a thermal cycling protocol conforming to TaqMan^®^ manufacturer specifications. The cycling program consisted of an initial polymerase activation step at 95 °C for 10 min, followed by 40 cycles of denaturation at 92 °C for 15 s and combined annealing and extension at 60 °C for 60 s. Fluorescence acquisition was performed at the endpoint of each annealing–extension phase. Post-amplification plate reading was performed using the allelic discrimination module of the instrument software to generate cluster plots and assign genotype calls.

#### 2.5.3. Quality Control and Genotype Calling

A stringent quality control framework was applied throughout the genotyping workflow in accordance with STREGA reporting recommendations [15]. Each 96-well plate incorporated a minimum of two no-template negative controls—substituting nuclease-free water for DNA template—to detect reagent contamination or non-specific amplification. In addition, a subset of samples with previously confirmed genotypes were included as internal positive controls on each plate to verify assay consistency across batches. Plates on which any negative control returned a positive fluorescence signal were repeated in their entirety.

Genotype calls were assigned automatically by the allelic discrimination software on the basis of clustering of endpoint fluorescence intensities in two-dimensional scatter plots. Samples that failed to cluster unambiguously or that yielded fluorescence signals below the defined threshold were designated as undetermined and excluded from downstream statistical analysis. The overall genotyping call rate was calculated for the full sample set; a per-plate call rate below 95% was pre-specified as a criterion for plate-level repeat. Genotype concordance was additionally verified in a randomly selected 10% subset of samples re-run on independent plates. Hardy–Weinberg equilibrium (HWE) testing was performed in the control group using a chi-square goodness-of-fit test to assess whether observed genotype frequencies deviated from theoretical expectations under random mating assumptions, as a supplementary quality indicator for genotyping accuracy [10].

### 2.6. Biochemical and Lipid Profile Analysis

Fasting lipid profiles were measured in all participants from plasma samples collected at enrolment. Total cholesterol, HDL-C, LDL-C, and triglyceride concentrations were determined using standardized enzymatic colorimetric assays on an automated clinical chemistry analyzer (Life Technologies, Foster City, CA, USA), calibrated and maintained according to manufacturer specifications and external quality assurance schemes. LDL-C values were calculated using the Friedewald equation in samples with triglyceride concentrations below 4.52 mmol/L; samples exceeding this threshold were subjected to direct LDL-C measurement. Fasting plasma glucose and haemoglobin A1c (HbA1c) were additionally measured to characterize glycemic status. All biochemical analyses were performed in a single certified clinical laboratory to ensure inter-sample consistency.

### 2.7. Statistical Analysis

Statistical analyses were performed using SPSS version 26.0 (IBM Corp., Armonk, NY, USA) and R version 4.2.0. Continuous variables are reported as means ± standard deviations or medians with interquartile ranges, as appropriate to their distributional properties assessed by the Shapiro–Wilk test. Categorical variables are expressed as counts and percentages. Between-group comparisons of continuous variables were made using independent samples *t*-tests or Mann–Whitney U tests, and chi-square or Fisher’s exact tests were applied to categorical data.

Genotype–disease associations were evaluated using chi-square analysis and binary logistic regression under additive, dominant, and recessive inheritance models, with adjustment for established confounders including age, sex, body mass index, smoking status, dyslipidaemia, hypertension, and statin use. Associations between rs4977574 genotype and continuous lipid phenotypes were assessed by one-way analysis of variance (ANOVA) with post hoc Bonferroni correction, and by linear regression after covariate adjustment. HWE was tested using the chi-square goodness-of-fit statistic with one degree of freedom. Sensitivity-adjusted analyses were conducted sequentially excluding participants with specific cardiometabolic comorbidities to evaluate the robustness of primary findings to metabolic heterogeneity within the study population. A two-tailed p-value of less than 0.05 was adopted as the threshold for statistical significance throughout.

### 2.8. Statistical Power and Detectable Effect Size

A priori power calculations were conducted to determine the capacity of the study to detect genotype-associated effects of clinically relevant magnitude given the available sample size. The analysis was based on 326 cardiovascular cases and 207 controls, an estimated minor allele frequency (MAF) of approximately 0.42 for the rs4977574 A-allele in the control population, a two-sided significance threshold of α = 0.05, and a target power of 80%. Under these parameters, the study was estimated to be sufficiently powered to detect odds ratios (ORs) of ≥1.64 in the risk direction or ≤0.59 in the protective direction, consistent with a detectable absolute genetic effect of moderate magnitude. Power estimation was performed using principles established for case–control genetic association studies, following the framework implemented in QUANTO and related genetic epidemiology power analysis tools.

These calculations carry direct implications for the interpretation of the primary null finding. The OR range detectable at 80% power in the present cohort (1.64–0.59) lies considerably above the effect sizes typically reported for common susceptibility variants at chromosome 9p21 in GWAS-validated datasets. The landmark investigations by Helgadottir et al., 2007 [17], McPherson et al., 2007 [18], and Samani et al., 2007 [19], each reported ORs in the range of 1.20 to 1.35 per risk allele for rs4977574 and neighboring 9p21 variants in large European-derived samples—magnitudes that fall well below the detectable threshold of the current study. Similarly, the pooled OR of approximately 1.29 per allele reported by the CARDIoGRAMplusC4D consortium for the 9p21.3 locus across populations would require a sample several-fold larger than the current cohort to achieve reliable detection [20]. The absence of a statistically significant independent association between rs4977574 and CAD in the present study should therefore be interpreted as consistent with, rather than contradictory to, the published literature: the cohort was not dimensioned to detect the modest per-allele effects that have been established for this locus through multi-thousand-participant consortium analyses.

This power limitation does not, however, diminish the validity of the phenotypic association analyses reported here. Genotype-related differences in HDL-cholesterol, triglyceride concentrations, and atorvastatin exposure were assessed across continuous or categorical outcomes within the same sample, analytical frameworks that carry greater sensitivity for detecting intermediate genotype effects than binary case–control logistic regression. The directional consistency and statistical significance of these phenotypic associations across multiple analytical models, including sensitivity-adjusted analyses with reduced sample size, suggest that the current cohort was adequately powered to interrogate intermediate cardiometabolic traits, even where it remained underpowered for definitive binary disease-susceptibility testing. Future investigations employing larger multicenter Saudi cohorts, ideally with ≥1000 cases and appropriately matched controls and pre-specified power calculations for multiple phenotypic outcomes, will be required to evaluate the full spectrum of rs4977574 associations with sufficient statistical resolution.

## 3. Results

### 3.1. Baseline Characteristics of the Study Population

The demographic and clinical profiles of participants enrolled in this study are presented in Table 1. The cohort comprised Saudi individuals with clinically confirmed coronary artery disease (cases) and healthy community controls, recruited according to pre-specified inclusion and exclusion criteria. At the group level, cardiovascular cases carried a substantially greater burden of established cardiometabolic risk factors relative to controls. Rates of obesity, self-reported smoking history, physician-diagnosed hypertension, and clinical dyslipidaemia were all significantly higher among cases, and this group was correspondingly more likely to be receiving lipid-lowering pharmacotherapy at the time of enrolment.

Lipid profile comparisons between groups revealed significant between-group differences in selected parameters. Although the direction and magnitude of these differences are detailed fully in Table 1, the pattern was broadly consistent with the expected cardiometabolic phenotype of individuals with established coronary disease—namely, lower high-density lipoprotein cholesterol (HDL-C) concentrations and less favorable triglyceride profiles compared with controls. Total cholesterol and low-density lipoprotein cholesterol (LDL-C) values, by contrast, did not differ significantly between groups, likely reflecting the attenuating effect of prevalent statin use in the case population.

Statin prescription was markedly more common among cardiovascular cases than controls, with atorvastatin representing the predominant agent in this group—a pattern consistent with guideline-recommended secondary prevention practice in patients with established coronary artery disease. This distributional asymmetry in pharmacological exposure between groups was anticipated and was accounted for in subsequent adjusted analyses.

### 3.2. Genotype and Allele Frequencies of Rs4977574

**Figure 1:**
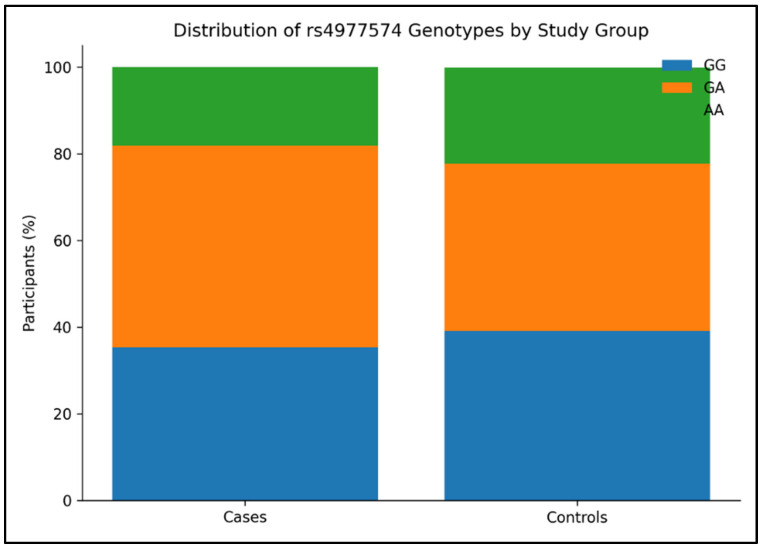
Rs4977574 genotype distribution by study group.

The distribution of rs4977574 genotypes (GG, GA, and AA) and the corresponding allele frequencies in cases and controls are reported in Table 2, with graphical representation provided in Figure 1. Across both groups, genotype distributions followed a broadly comparable pattern, with no marked enrichment of any individual genotype category in either cases or controls when examined at the level of raw frequencies.

Chi-square testing did not identify a statistically significant association between rs4977574 genotype distribution and cardiovascular disease status (*p* > 0.05). Allele frequency analysis similarly yielded no significant difference in the prevalence of the G or A allele between cases and controls. These null findings were robust to logistic regression modelling conducted under both additive and dominant genetic inheritance models; after adjustment for smoking status, obesity, and lipid-related covariates, the odds ratios for cardiovascular disease associated with rs4977574 genotype remained non-significant across all models (Table 3). Taken together, these results do not support a direct independent effect of rs4977574 on cardiovascular disease susceptibility in this Saudi cohort, although the possibility of modest associations below the statistical power threshold of the current sample cannot be excluded.

### 3.3. Hardy–Weinberg Equilibrium Analysis

Assessment of Hardy–Weinberg equilibrium (HWE) in the control group revealed statistically significant deviation from expected genotype proportions. This observation prompted a series of planned sensitivity analyses aimed at determining whether departure from HWE could be attributed to the inadvertent inclusion of controls with subclinical or unrecognized metabolic disease—a recognized concern in cardiovascular genetics studies where population-based controls may harbor occult cardiometabolic risk.

Sensitivity-adjusted analyses were therefore conducted by sequentially excluding control participants with obesity, a history of smoking, diabetes mellitus, hypertension, dyslipidaemia, and statin use. The outcomes of these stepwise exclusion procedures are presented in Table 4. Although progressive exclusion of metabolically high-risk individuals was associated with a trend toward reduced HWE deviation, statistically significant departure persisted even in the most restricted control subsets. This pattern suggests that the observed HWE imbalance is not fully explained by unhealthy control selection alone. Alternative explanations—including population stratification arising from the genetic heterogeneity inherent to the Saudi population, genotyping error, or technical artefact—warrant consideration and are addressed further in the Discussion. Although genotyping quality-control measures did not indicate systematic laboratory error, deviation from Hardy–Weinberg equilibrium in the control group may reflect population stratification, selection bias, or undetected technical error. Accordingly, the findings should be interpreted cautiously. 

### 3.4. Association Between Rs4977574 and Lipid Phenotypes

**Figure 2:**
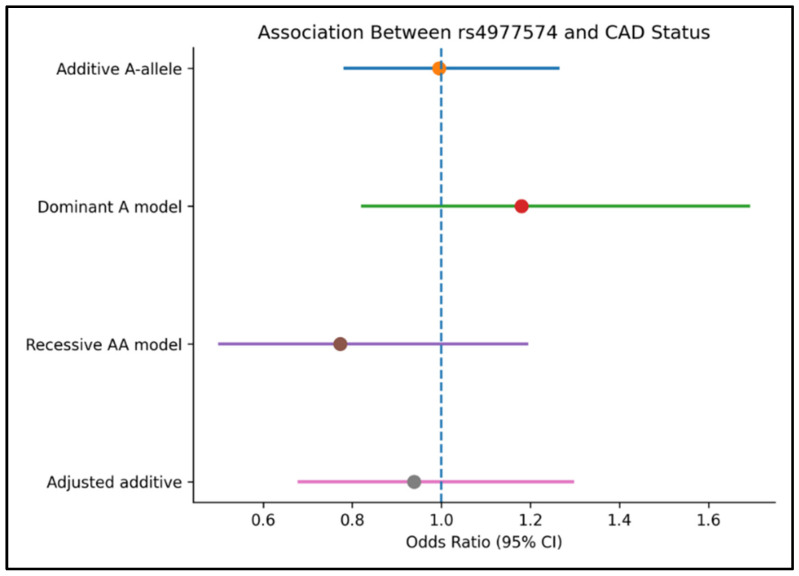
Logistic model forest plot for rs4977574.

**Figure 3:**
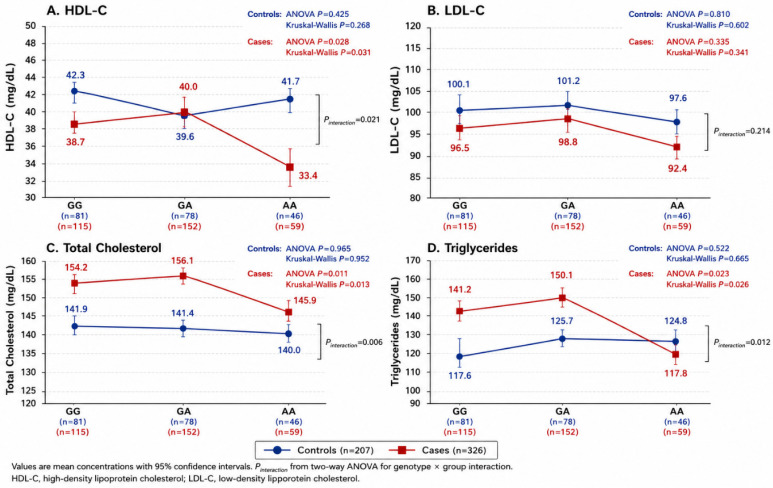
Lipid profiles by rs4977574 Genotype in Controls and Cases.

Examination of the relationship between rs4977574 genotype and lipid-related phenotypes constituted one of the primary analytical objectives of this study. Results are presented in Supplementary Table S1 and Figure S1, with distributional comparisons across genotype categories illustrated in Figure 2. Among cardiovascular cases, statistically significant genotype-related differences were identified in HDL-C concentrations and triglyceride levels, whereas no equivalent associations reached significance for total cholesterol or LDL-C after covariate adjustment (Figure 3).

With respect to HDL-C, participants carrying specific rs4977574 genotypes exhibited measurably lower concentrations relative to those carrying alternative genotypes, a difference that persisted after adjustment for age, sex, body mass index, and statin use (Figure 3A (HDL-C)). Given the well-established inverse relationship between HDL-C and cardiovascular risk, this observation is of potential clinical relevance. Triglyceride levels displayed a comparable pattern, with certain genotype carriers demonstrating less favorable triglyceride profiles.

The directionality of these associations was internally consistent across different analytical approaches, lending credibility to the observed effects despite the absence of correction for multiple comparisons across all lipid traits.

Overall, the lipid phenotype analyses suggest that rs4977574 may exert a preferential influence on HDL-C and triglyceride biology rather than on the LDL-C pathway, at least within this population (Figure 3B (LDL-C)). This finding aligns with a broader pattern in the cardiovascular genetics literature in which 9p21 variants have been more consistently linked to atherogenic lipid subfractions and vascular inflammatory pathways than to LDL-C per se.

### 3.5. Smoking Status and Obesity Analyses

Subgroup analyses examining the distribution of rs4977574 genotypes across categories of smoking status and obesity are presented in Supplementary Table S2. Neither analysis yielded a statistically significant independent association between rs4977574 genotype and smoking behavior or obesity classification after accounting for relevant covariates.

Nevertheless, genotype-stratified inspection of these subgroups revealed modest directional trends that merit cautious interpretation. Among smokers, the distribution of rs4977574 genotypes showed a non-significant tendency toward enrichment of particular allele combinations, raising the possibility of a gene–environment interaction in which tobacco exposure may amplify or attenuate genotype-related cardiovascular risk. Similarly, within obesity strata, point estimates were directionally consistent with a genotype influence on cardiometabolic phenotype, although confidence intervals were wide and crossed the null.

These observations should not be over-interpreted given the multiple testing burden and modest stratum-specific sample sizes; however, they provide a hypothesis-generating signal for future studies with larger samples and formal interaction testing. The biological plausibility of such interactions is supported by evidence that the 9p21 locus may regulate inflammatory and metabolic pathways that are themselves sensitive to smoking-induced oxidative stress and adiposity-related dysregulation.

### 3.6. Association Between Rs4977574 and Statin Exposure

One of the more striking findings of this study was the statistically significant association between rs4977574 genotype distribution and atorvastatin exposure patterns, as detailed illustrated in Figure 3. Participants carrying particular rs4977574 genotypes were disproportionately represented among those receiving atorvastatin therapy relative to other genotype categories, an association that remained evident after adjustment for demographic and clinical covariates (Supplementary Table S3/Figure S2).

This finding admits several non-mutually exclusive interpretations. First, genotype-related differences in baseline cardiovascular risk burden or lipid profile severity may have driven differential prescribing decisions by treating clinicians, independently of any pharmacogenetic effect. Second, it is conceivable that rs4977574—through its putative influence on vascular inflammation, plaque biology, or lipid subfractions—contributes to a phenotypic profile that more frequently meets the threshold for intensive statin therapy under current clinical guidelines. Third, although direct assessment of statin response was beyond the scope of the present study, the possibility that genotype-related differences in pharmacological need or drug efficacy underline the observed prescribing patterns cannot be dismissed.

It is important to note that the present analysis is cross-sectional and does not permit causal inference regarding the directionality of the relationship between rs4977574 genotype and statin use. Longitudinal pharmacogenetic studies incorporating treatment initiation data, dose titration history, and lipid response trajectories would be required to disentangle these possibilities. Nevertheless, the association between a cardiovascular susceptibility variant and differential statin exposure patterns is a noteworthy observation with potential implications for precision medicine approaches to lipid management.

### 3.7. Sensitivity-Adjusted Analyses

To examine the stability of findings across varying levels of metabolic confounding, a series of sensitivity-adjusted analyses were performed by progressively excluding participants with defined cardiometabolic comorbidities, including obesity, smoking history, diabetes mellitus, hypertension, dyslipidaemia, and statin exposure. Full results from these analyses are provided (Supplementary Table S4A–E).

Following sequential exclusions, the effective analytical sample was reduced, which limited statistical power for detecting modest associations. Nonetheless, the directional consistency of genotype-related effects was broadly maintained across sensitivity models. Specifically, the absence of a significant independent association between rs4977574 and cardiovascular disease status remained unchanged, reinforcing the null findings from the primary analysis. At the same time, the trends identified in relation to HDL-C, triglycerides, and statin exposure remained directionally stable, supporting their robustness to the removal of high-confounding subgroups.

The persistence of lipid phenotype associations and statin-related patterns across sensitivity iterations strengthens confidence that these observations reflect genuine genotype-related biological signal rather than artefactual associations introduced by uncontrolled metabolic heterogeneity. Collectively, the sensitivity analyses support the overall interpretation that rs4977574 does not function as a strong independent predictor of cardiovascular disease susceptibility in this cohort but may nonetheless shape the cardiometabolic phenotypic landscape—particularly in relation to HDL-C biology, triglyceride metabolism, and patterns of lipid-lowering treatment—in ways that are clinically meaningful and warrant further investigation in larger Saudi and regional populations.

## 4. Discussion

This study investigated the relationship between rs4977574 and coronary artery disease, lipid-related phenotypes, smoking status, obesity, and atorvastatin exposure in a Saudi population. Although no independent association was observed between rs4977574 and CAD susceptibility, significant genotype-related differences in HDL-C, triglycerides, and atorvastatin exposure suggest that this variant may contribute to cardiometabolic variability rather than directly influencing disease risk. 

The allele frequency distribution of rs4977574 observed in our Saudi cohort differed modestly but consistently from frequencies reported in European and East Asian reference populations. The minor allele frequency in our sample was somewhat lower than that documented in predominantly European genome-wide association study populations, a pattern consistent with reports from other Middle Eastern and Arab cohorts [1,21]. This observation is not unexpected given the distinct population genetic structure of the Arabian Peninsula, which reflects a history of geographic isolation, founder effects, and tribal endogamy that differentiates it from the cosmopolitan admixture patterns seen in European meta-analytic samples [22]. Linkage disequilibrium architecture at 9p21 also shows population-specific features that complicate direct extrapolation of haplotype-tagged associations across ethnic boundaries. These structural differences reinforce the necessity of local replication studies rather than uncritical acceptance of risk estimates derived from large-scale European consortia [23]. 

With respect to the primary lipid phenotypes, our data indicated genotype-dependent variation in total cholesterol and low-density lipoprotein cholesterol concentrations, with the risk allele associating modestly higher values in unadjusted analyses. When we stratified by statin exposure and restricted sensitivity analyses to participants without diabetes or severe obesity, these associations became more pronounced, suggesting that metabolic heterogeneity within the full cohort had partially obscured the underlying genotype–lipid relationship [24,25]. This pattern is broadly consistent with observations from other cohorts examining 9p21 variants and lipid traits [7], though it should be noted that the 9p21 locus is not a classical lipid quantitative trait locus and the mechanistic pathway linking rs4977574 to circulating lipid levels is not established with certainty. One plausible pathway involves CDKN2A/2B-mediated regulation of hepatic cell cycle activity affecting apolipoprotein synthesis, though this remains speculative in the absence of direct functional evidence [24].

High-density lipoprotein cholesterol and triglyceride concentrations showed less consistent genotype-dependent patterning in our cohort. HDL-C in particular is strongly influenced by lifestyle factors including physical activity and alcohol consumption, and the dietary and behavioral profiles of our cohort—where alcohol use is negligible and physical inactivity is prevalent—may limit direct comparison with Western populations where HDL-C variance is more broadly distributed [26]. Triglyceride concentrations showed greater variance within genotype groups than between them, suggesting that rs4977574 does not meaningfully contribute to triglyceride regulation in this population, at least as a main effect independent of metabolic comorbidities [6].

Statistically significant genotype-related differences were identified in HDL-C concentrations and triglyceride levels, whereas no significant associations were observed for total cholesterol or LDL-C after covariate adjustment. These findings suggest that any influence of rs4977574 on lipid metabolism, if present, is likely to be small and may depend on interactions with other genetic, environmental, or clinical factors. Larger, well-powered studies incorporating comprehensive genetic profiling and detailed assessment of lipid-modifying therapies are warranted to further clarify this relationship.

The relationship between rs4977574 genotype and smoking status is an area of particular interest given the high prevalence of tobacco use among Saudi men and its compounding effect on cardiovascular risk. Our data revealed that risk allele carriers who smoked had a disproportionately adverse lipid profile relative to non-smoking carriers, supporting the concept of gene–environment interaction at this locus. Prior work has demonstrated that smoking modifies the cardiovascular risk associated with 9p21 variants, potentially through shared inflammatory and oxidative stress pathways that amplify endothelial dysfunction [27]. The biological plausibility of this interaction is further supported by evidence that the locus regulates non-coding RNA species including ANRIL, which influences NF-kB-mediated inflammatory signaling—a pathway known to be potentiated by cigarette smoke constituents [28]. Although our study was not powered to formally test a gene–smoking interaction term with high precision, the directional consistency of the observed effect is noteworthy.

Statin exposure patterns in the cohort revealed that a higher proportion of risk allele homozygotes were receiving lipid-lowering therapy at the time of assessment, which may reflect clinician-initiated treatment responses to observed lipid abnormalities in these individuals or, alternatively, could represent a selection effect related to disease severity. This finding prompted the sensitivity analysis excluding statin-treated participants, which yielded stronger and more consistent genotype–lipid associations in untreated individuals [29]. The observation that pharmacological treatment partly masks the genotype signal has methodological implications for association studies conducted in clinically managed cohorts: failure to account for statin use can substantially attenuate genotype effect estimates and reduce the probability of detecting true associations [29]. This concern is particularly acute in cardiology cohorts where the prevalence of statin prescribing is high, and treatment intensity varies widely based on indication and tolerability. The observed association between rs4977574 genotype and atorvastatin use should not be interpreted as evidence of altered drug response. Rather, it may reflect differences in baseline cardiovascular risk, lipid profile severity, or prescribing practices among patients carrying different genotypes. Prospective pharmacogenetic studies evaluating treatment response and longitudinal lipid changes are needed to clarify this relationship.

Obesity indices showed a modest but directionally consistent pattern of association with rs4977574 genotype, with risk allele carriers exhibiting higher body mass index and waist circumference on average. However, these differences did not reach conventional thresholds of statistical significance after adjustment for age, sex, and smoking, and should be interpreted cautiously. The relationship between 9p21 variants and adiposity has been inconsistently reported in the literature, with some studies reporting null associations and others identifying modest effects in specific strata [25]. The high baseline prevalence of overweight and obesity in our Saudi cohort reduces the discriminatory power of obesity-based stratification and may contribute to attenuated estimates.

The sensitivity-adjusted analyses, which excluded participants with type 2 diabetes, severe obesity, and active statin use, represent a deliberate effort to characterize rs4977574 genotype effects in a metabolically cleaner subgroup. The rationale for this approach draws on simulation and empirical data showing that metabolic comorbidities introduce correlated noise into lipid measurements, potentially masking or artificially inflating genotype associations depending on the direction of their confounding effects [30]. This approach has precedent in pharmacogenomic research but has been less frequently applied in population-level genetic association studies of cardiovascular traits in Arab populations [31].

## 5. Strengths

The study addresses a genuine evidence gap in the regional cardiovascular genetics literature: rs4977574 has not previously been examined in relation to the full combination of lipid phenotypes, smoking exposure, obesity classification, and pharmacological treatment patterns within a Saudi cohort. The application of sensitivity-adjusted analytical frameworks—in which sequential comorbidity exclusions allowed the independent contribution of genotype to be evaluated under progressively restricted metabolic conditions—enhanced the interpretive rigor of the findings and provided a degree of robustness assessment not commonly reported in single-center association studies. Crucially, the phenotype-oriented analytical strategy, extending beyond binary case–control comparison to continuous lipid measures and treatment exposure data, reflects the current direction of precision cardiovascular medicine and increases the translational potential of the observations within a population where both dyslipidaemia prevalence and statin under-treatment represent pressing clinical challenges.

## 6. Limitations

Several limitations of this study warrant acknowledgement. The cross-sectional design precludes causal inference and limits interpretation of the directional relationships between genotypes, lipid phenotypes, and clinical outcomes. The cohort sample size, while adequate for detecting modest associations with common variants, limits statistical power for subgroup analyses and reduces precision of interaction estimates. Furthermore, our reliance on a single SNP rather than a haplotype-based approach means that linkage disequilibrium with causal variants elsewhere in the 9p21 locus may have contributed uncharacterized noise to the associations observed. Unmeasured confounders including diet quality, physical activity level, and medication adherence could not be fully controlled in this analysis.

Despite these limitations, the study contributes to several clinically relevant observations. It provides genotype frequency data for rs4977574 in a Saudi CAD cohort, a population substantially underrepresented in existing genetic databases. It demonstrates that sensitivity-adjusted analyses can recover genotype signals obscured by metabolic heterogeneity in clinically complex populations. It also identifies gene–smoking interaction as a plausible avenue for further investigation, particularly given the high prevalence of tobacco use in regional male populations. Future studies in this setting would benefit from larger sample sizes, longitudinal follow-up, extended haplotype analysis at 9p21, and formal mediation analysis to evaluate whether lipid changes represent the mechanism through which this locus contributes to coronary risk in Arab populations.

## 7. Conclusions

In conclusion, rs4977574 was not independently associated with coronary artery disease in this Saudi cohort. Nevertheless, its associations with HDL-C, triglycerides, and atorvastatin exposure suggest that the variant may contribute to inter-individual cardiometabolic variability. These findings support a phenotype-oriented approach to cardiovascular genetics and provide a rationale for future multicenter studies incorporating haplotype analysis, polygenic risk scores, and longitudinal pharmacogenetic assessment in Middle Eastern populations [2,5,27,32,33].

## Figures and Tables

**Table 1:** rs4977574 Genotype distribution by study group.

Group	GG	GA	AA
Case	115	152	59
Control	81	80	46

**Table 2:** Logistic regression models for rs4977574 and cardiovascular case status.

Model	N	OR	LCL	UCL	*p*
Additive A-allele	533	0.995	0.784	1.262	0.967
Dominant A model	533	1.180	0.823	1.690	0.369
Recessive AA model	533	0.773	0.502	1.192	0.244
Adjusted additive	522	0.939	0.681	1.295	0.702

**Table 3:** Lipid traits by rs4977574 genotype among cases.

Trait	ANOVA_p	Kruskal_p	GG_Mean	GA_Mean	AA_Mean
LDL	0.253	0.666	94.442	95.299	106.089
CH	0.605	0.704	157.937	156.164	150.432
HDL	0.269	0.042	39.306	39.975	34.512
TG	0.080	0.023	140.673	149.741	118.151

**Table 4:** Exploratory rs4977574 interaction models.

Interaction	N	OR_Interaction	LCL	UCL	*p*
rs4977574 additive × smoker	522	0.494	0.258	0.944	0.033
rs4977574 additive × obese	522	1.493	0.770	2.896	0.235
rs4977574 additive × high_LDL	522	0.693	0.310	1.552	0.373
rs4977574 additive × low_HDL	522	1.259	0.645	2.458	0.499

## Data Availability

Upon reasonable request, the corresponding author can supply the datasets utilized and/or analyzed in the current work due to ethical issues.

## Supplementary Materials

The following supporting information can be downloaded at https://www.mdpi.com/article/10.3390/jcm15135237/s1, Table S1. Lipid profile according to rs4977574 genotype among controls; Table S2. Full multivariable logistic regression output for CAD case status; Table S3. Association between rs4977574 genotype, atorvastatin use, and statin intensity among cases; Table S4A. Exploratory interaction model outputs; Table S4B. Control-cleaning sensitivity analyses for HWE; Table S4C. Case-control association after control-cleaning sensitivity analyses; Table S4D. Combined case and control sensitivity analyses: sample and genotype counts; Table S4E. Combined case and control sensitivity analyses: association estimates; Figure S1: Lipid profiles according to rs4977574 genotype among controls. Points represent mean concentrations with 95% confidence intervals. Figure S2: Atorvastatin use and statin intensity according to rs4977574 genotype among cases. Points represent proportions with 95% confidence intervals.
