# Supplementary material for: Association of rs4977574 with Lipid Phenotypes, Smoking Status, and Statin Exposure in a Saudi Cardiovascular Cohort: A Sensitivity-Adjusted Genetic Association Study
Source: J Clin Med. 2026 Jul 4;15(13):5237. doi: 10.3390/jcm15135237 (PMC13363314; doi:10.3390/jcm15135237)
Supplement: Supplementary file 1 [file jcm-15-05237-s001.zip › jcm-4379313-supplementary.pdf]

Supplementary Tables and Figures

Supplementary Table S1. Lipid profile according to rs4977574 genotype among controls

Values are presented as mean ± SD. Zero lipid values were treated as missing where applicable. P values were calculated using one-way ANOVA and Kruskal-Wallis tests.

Lipid parameter	GG	GA	AA	ANOVA P	Kruskal-Wallis P
HDL-C	42.3 ± 12.4 (n=81)	39.6 ± 11.5 (n=78)	41.7 ± 17.9 (n=46)	0.425	0.268
LDL-C	100.1 ± 30.3 (n=81)	101.2 ± 30.7 (n=78)	97.6 ± 27.7 (n=46)	0.810	0.602
Total cholesterol	141.9 ± 38.0 (n=81)	141.4 ± 40.3 (n=77)	140.0 ± 40.4 (n=46)	0.965	0.952
Triglycerides	117.6 ± 36.4 (n=81)	125.7 ± 47.9 (n=78)	124.8 ± 61.9 (n=46)	0.522	0.665

Abbreviations: HDL-C, high-density lipoprotein cholesterol; LDL-C, low-density lipoprotein cholesterol; SD, standard deviation.

Supplementary Table S2. Full multivariable logistic regression output for CAD case status

This model uses complete available observations with age, sex/gender code, BMI, smoking history, statin exposure, LDL-C, HDL-C, triglycerides, and additive rs4977574 A-allele count. Interpret covariates according to the coding used in the supplied dataset.

Term	N	B	SE	Wald chi-square	OR	95% CI lower	95% CI upper	p-value
const	501	-4.241	1.027	17.063	0.014	0.002	0.108	<0.001
A_count	501	-0.051	0.178	0.083	0.950	0.671	1.346	0.773
age	501	0.090	0.010	79.766	1.094	1.073	1.116	<0.001
gender	501	-0.545	0.312	3.045	0.580	0.314	1.069	0.081
BMI	501	-0.011	0.012	0.878	0.989	0.965	1.013	0.349
Current_or_previous_smoker	501	0.508	0.311	2.668	1.662	0.903	3.059	0.102
Any_statin	501	1.980	0.304	42.398	7.243	3.991	13.146	<0.001
LDL	501	-0.001	0.004	0.032	0.999	0.992	1.007	0.858
HDL	501	0.005	0.010	0.233	1.005	0.986	1.024	0.629
TG	501	0.003	0.002	1.835	1.003	0.999	1.007	0.176

Abbreviations: BMI, Body mass index; HDL-C, high-density lipoprotein cholesterol; LDL-C, low-density lipoprotein cholesterol

Supplementary Table S3. Association between rs4977574 genotype, atorvastatin use, and statin intensity among cases

Values are presented as n (%). High-intensity atorvastatin was defined as 40/80 mg, and moderate-intensity atorvastatin was defined as 10/20 mg.

Genotype	Total N	Atorvastatin use, n (%)	High-intensity atorvastatin, n (%)	Moderate-intensity atorvastatin, n (%)
GG	115	65 (56.5)	47 (40.9)	18 (15.7)
GA	152	96 (63.2)	79 (52.0)	16 (10.5)
AA	59	30 (50.8)	22 (37.3)	8 (13.6)

Statistical comparison across genotype groups:

Analysis	Chi-square	P value
Atorvastatin use	2.968	0.227
High-intensity atorvastatin	5.168	0.075
Moderate-intensity atorvastatin	1.562	0.458

Supplementary Table S4A. Exploratory interaction model outputs

Interaction terms are exploratory and should be interpreted cautiously because no formal multiple-testing correction was applied.

Interaction term	N	B	SE	Wald chi-square	OR	95% CI lower	95% CI upper	p-value
rs4977574 additive × smoker	522	-0.705	0.330	4.555	0.494	0.258	0.944	0.033
rs4977574 additive × obese	522	0.401	0.338	1.409	1.493	0.770	2.896	0.235
rs4977574 additive × high_LDL	522	-0.366	0.411	0.794	0.693	0.310	1.552	0.373
rs4977574 additive × low_HDL	522	0.231	0.341	0.457	1.259	0.645	2.458	0.499

Hardy-Weinberg equilibrium and sensitivity analyses

Supplementary Table S4B. Control-cleaning sensitivity analyses for HWE

HWE was evaluated in controls across sequential exclusion scenarios. Persistent deviation after metabolic exclusions suggests that the imbalance was not explained solely by unhealthy control selection.

Scenario	Controls	Removed_controls	GG	GA	AA	Missing_genotype	HWE_exact_p
Original controls	251	0.000	81.000	80.000	46.000	44.000	0.004
Remove controls with HTN/DM only	183	68.000	65.000	56.000	35.000	27.000	0.002
Remove controls with HTN/DM/PCVD/stroke/HF	146	105	46.000	44.000	32.000	24.000	0.003
Remove current/previous smokers only	156	95.000	64.000	40.000	29.000	23.000	<0.001
Remove obese controls only	174	77.000	56.000	47.000	40.000	31.000	<0.001
Remove abnormal LDL only	226	25.000	73.000	72.000	40.000	41.000	0.010
Remove low HDL only	112	139	36.000	28.000	17.000	31.000	0.018
Remove lipid abnormal categories only	87.000	164	26.000	20.000	15.000	26.000	0.016
Remove metabolic risk: smoking/obesity/lipid	42.000	209	16.000	7.000	10.000	9.000	0.001
Remove strict requested risks	28.000	223	9.000	5.000	8.000	6.000	0.012

Supplementary Table S4C. Case-control association after control-cleaning sensitivity analyses

OR = odds ratio for the additive A-allele model.

Scenario	Genotype_case_control_p	Additive_OR	Additive 95% CI	Additive_p
Original controls	0.178	0.995	0.784-1.262	0.967
Remove controls with HTN/DM only	0.083	1.039	0.800-1.349	0.773
Remove controls with HTN/DM/PCVD/stroke/HF	0.071	0.900	0.678-1.194	0.464
Remove current/previous smokers only	0.004	1.186	0.899-1.565	0.228
Remove obese controls only	0.009	0.897	0.689-1.169	0.422
Remove abnormal LDL only	0.233	1.013	0.790-1.297	0.922
Remove low HDL only	0.143	1.129	0.804-1.585	0.485
Remove lipid abnormal categories only	0.128	1.016	0.697-1.483	0.933
Remove metabolic risk: smoking/obesity/lipid	0.017	1.019	0.622-1.670	0.940
Remove strict requested risks	0.042	0.788	0.436-1.422	0.429

Supplementary Table S4D. Combined case and control sensitivity analyses: sample and genotype counts

This table shows how the genotype counts changed after case and control restrictions.

Scenario	N_cases	N_controls	case_GG	case_GA	case_AA	control_GG	control_GA	control_AA	HWE_controls_p
Original all cases + all controls	326	207	115	152	59.000	81.000	80.000	46.000	0.004
Case-clean strict only; all controls	65.000	207	24.000	32.000	9.000	81.000	80.000	46.000	0.004
Case-clean broad only; all controls	24.000	207	8.000	11.000	5.000	81.000	80.000	46.000	0.004
Controls no HTN/DM + all cases	326	156	115	152	59.000	65.000	56.000	35.000	0.002
Controls no HTN/DM + case-clean strict	65.000	156	24.000	32.000	9.000	65.000	56.000	35.000	0.002

Controls no HTN/DM + case-clean broad	24.000	156	8.000	11.000	5.000	65.000	56.000	35.000	0.002
Controls clinical clean + case-clean strict	65.000	122	24.000	32.000	9.000	46.000	44.000	32.000	0.003
Controls metabolic borderline clean + case-clean broad	24.000	27.000	8.000	11.000	5.000	13.000	6.000	8.000	0.006

Supplementary Table S4E. Combined case and control sensitivity analyses: association estimates

These analyses show that reducing selected cases and controls did not materially alter the overall null association between rs4977574 and case-control status.

Scenario	genotype_chi2_p	allele_A_OR	allele_A_CI95	allele_p	additive_OR	additive_p	adjusted_OR_age_gender	adjusted_p_age_gender
Original all cases + all controls	0.178	0.994	0.774-1.277	1.000	0.995	0.967	0.928	0.643
Case-clean strict only; all controls	0.210	0.879	0.587-1.317	0.542	0.894	0.560	0.870	0.569
Case-clean broad only; all controls	0.783	1.094	0.599-2.000	0.760	1.079	0.788	1.191	0.591
Controls no HTN/DM + all cases	0.083	1.043	0.793-1.373	0.780	1.039	0.773	1.051	0.812
Controls no HTN/DM +	0.135	0.923	0.606-1.404	0.750	0.933	0.728	1.116	0.722

case-clean strict								
Controls no HTN/DM + case-clean broad	0.628	1.148	0.622-2.120	0.753	1.119	0.691	1.840	0.134
Controls clinical clean + case-clean strict	0.092	0.787	0.510-1.215	0.323	0.814	0.318	1.039	0.905
Controls metabolic borderline clean + case-clean broad	0.203	1.131	0.515-2.486	0.842	1.098	0.789	3.306	0.076

Supplementary Figure S1

Lipid profiles according to rs4977574 genotype among controls. Points represent mean concentrations with 95% confidence intervals.

Supplementary Figure S1. Lipid Profiles by rs4977574 Genotype in Controls

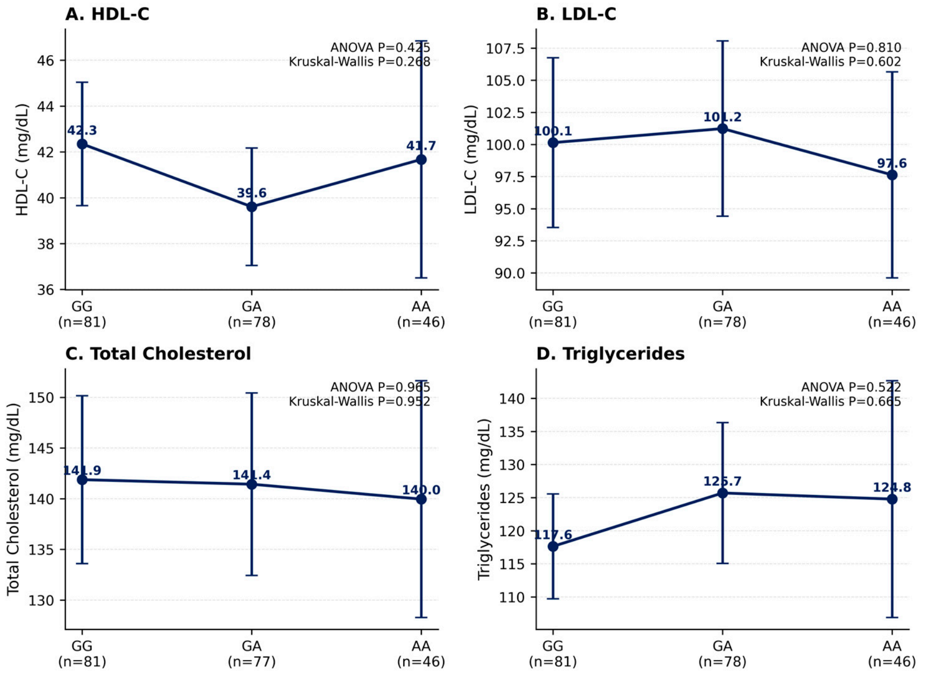

Points represent mean lipid concentrations with 95% confidence intervals among controls.

Supplementary Figure S2

Atorvastatin use and statin intensity according to rs4977574 genotype among cases. Points represent proportions with 95% confidence intervals.

Supplementary Figure S2. Atorvastatin Use and Statin Intensity by rs4977574 Genotype (Cases)

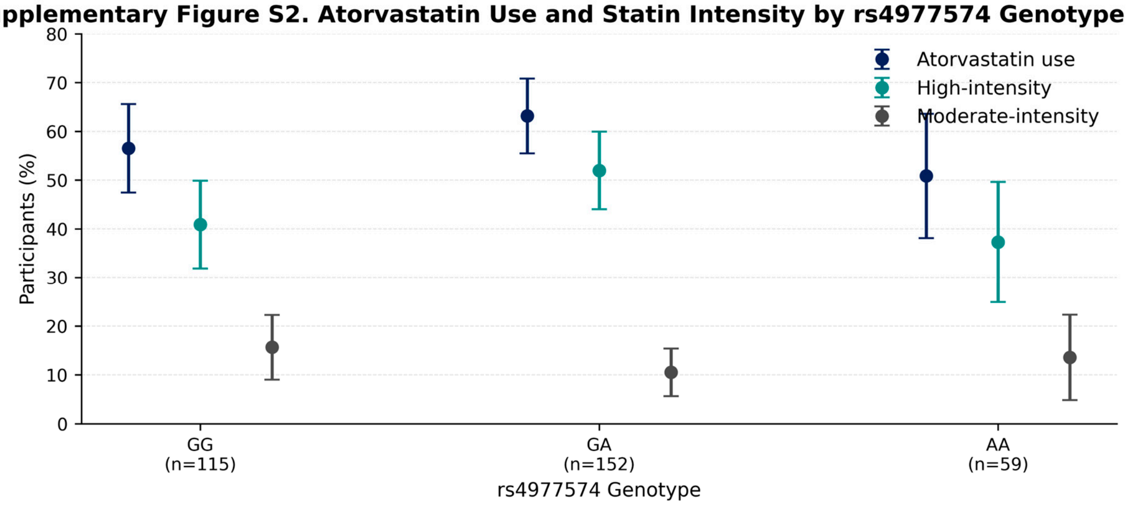

χ^2 P values: atorvastatin use=0.227; high-intensity=0.075; moderate-intensity=0.458

High-intensity: atorvastatin 40/80 mg; moderate-intensity: atorvastatin 10/20 mg.
